# SARS‐CoV‐2 infection and effects of age, sex, comorbidity, and vaccination among older individuals: A national cohort study

**DOI:** 10.1111/irv.13224

**Published:** 2023-11-21

**Authors:** Mai A. Mahmoud, Houssein H. Ayoub, Peter Coyle, Patrick Tang, Mohammad R. Hasan, Hadi M. Yassine, Asmaa A. Al Thani, Zaina Al‐Kanaani, Einas Al‐Kuwari, Andrew Jeremijenko, Anvar Hassan Kaleeckal, Ali Nizar Latif, Riyazuddin Mohammad Shaik, Hanan F. Abdul‐Rahim, Gheyath K. Nasrallah, Mohamed Ghaith Al‐Kuwari, Adeel A. Butt, Hamad Eid Al‐Romaihi, Mohamed H. Al‐Thani, Abdullatif Al‐Khal, Roberto Bertollini, Laith J. Abu‐Raddad, Hiam Chemaitelly

**Affiliations:** ^1^ Weill Cornell Medicine‐Qatar Cornell University Doha Qatar; ^2^ Mathematics Program Department of Mathematics, Statistics, and Physics College of Arts and Sciences Qatar University Doha Qatar; ^3^ Hamad Medical Corporation Doha Qatar; ^4^ Biomedical Research Center QU Health Qatar University Doha Qatar; ^5^ Wellcome‐Wolfson Institute for Experimental Medicine Queens University Belfast UK; ^6^ Department of Pathology Sidra Medicine Doha Qatar; ^7^ Department of Pathology and Molecular Medicine McMaster University Hamilton Canada; ^8^ Department of Biomedical Science College of Health Sciences QU Health Qatar University Doha Qatar; ^9^ Department of Public Health College of Health Sciences QU Health Qatar University Doha Qatar; ^10^ Primary Health Care Corporation Doha Qatar; ^11^ Department of Medicine Weill Cornell Medicine Cornell University New York New York USA; ^12^ Department of Population Health Sciences Weill Cornell Medicine Cornell University New York New York USA; ^13^ Ministry of Public Health Doha Qatar; ^14^ Infectious Disease Epidemiology Group Weill Cornell Medicine‐Qatar Cornell University Doha Qatar; ^15^ World Health Organization Collaborating Centre for Disease Epidemiology Analytics on HIV/AIDS Sexually Transmitted Infections, and Viral Hepatitis Weill Cornell Medicine–Qatar Cornell University, Qatar Foundation – Education City Doha Qatar; ^16^ College of Health and Life Sciences Hamad bin Khalifa University Doha Qatar

**Keywords:** COVID‐19, geriatrics, immunity, older adults, Qatar, vaccination

## Abstract

**Background:**

We investigated the contribution of age, coexisting medical conditions, sex, and vaccination to incidence of severe acute respiratory syndrome coronavirus 2 (SARS‐CoV‐2) infection and of severe, critical, or fatal COVID‐19 in older adults since pandemic onset.

**Methods:**

A national retrospective cohort study was conducted in the population of Qatar aged ≥50 years between February 5, 2020 and June 15, 2023. Adjusted hazard ratios (AHRs) for infection and for severe coronavirus disease 2019 (COVID‐19) outcomes were estimated through Cox regression models.

**Results:**

Cumulative incidence was 25.01% (95% confidence interval [CI]: 24.86–25.15%) for infection and 1.59% (95% CI: 1.55–1.64%) for severe, critical, or fatal COVID‐19 after a follow‐up duration of 40.9 months. Risk of infection varied minimally by age and sex but increased significantly with coexisting conditions. Risk of infection was reduced with primary‐series vaccination (AHR: 0.91, 95% CI: 0.90–0.93) and further with first booster vaccination (AHR: 0.75, 95% CI: 0.74–0.77). Risk of severe, critical, or fatal COVID‐19 increased exponentially with age and linearly with coexisting conditions. AHRs for severe, critical, or fatal COVID‐19 were 0.86 (95% CI: 0.7–0.97) for one dose, 0.15 (95% CI: 0.13–0.17) for primary‐series vaccination, and 0.11 (95% CI: 0.08–0.14) for first booster vaccination. Sensitivity analysis restricted to only Qataris yielded similar results.

**Conclusion:**

Incidence of severe COVID‐19 in older adults followed a dynamic pattern shaped by infection incidence, variant severity, and population immunity. Age, sex, and coexisting conditions were strong determinants of infection severity. Vaccine protection against severe outcomes showed a dose–response relationship, highlighting the importance of booster vaccination for older adults.

## 1 INTRODUCTION

The emergence of the severe acute respiratory syndrome coronavirus 2 (SARS‐CoV‐2) and the ensuing coronavirus disease 2019 (COVID‐19) pandemic resulted in millions of infections, hospitalizations, and deaths, with older populations being disproportionately affected by COVID‐19 severity.^1^, ^2^ The World Health Organization estimated that over 80% of COVID‐19 mortality during the years 2020 and 2021 occurred in individuals ≥60 years of age.^3^

Age‐related decrease in physiological reserve, functional capacity, and weakening of the immune response (immunosenescence) coupled with age‐related health conditions increase the susceptibility of older adults to symptomatic infection and progression to severe forms of COVID‐19.^4^, ^5^, ^6^, ^7^, ^8^, ^9^ Consequently, there has been an emphasis on prioritizing vaccination of older individuals as part of the vaccination campaigns.^3^ Current guidelines continue to recommend booster vaccination for older populations and individuals with coexisting medical conditions.^10^, ^11^

While the vulnerability of older individuals to this infection and its more severe forms is well‐established, there remains an inadequate understanding of the interplay *within* the older demographic, of infection and of predisposing factors including age, sex, pre‐existing health conditions, and the varying number of vaccine doses. Leveraging the national, federated platforms for SARS‐CoV‐2 infection and COVID‐19 vaccination in Qatar, this study aimed to investigate the role of age, coexisting conditions, sex, and vaccination in the incidence of SARS‐CoV‐2 infection and associated severe,^12^ critical,^12^ or fatal^13^ COVID‐19 in the national cohort of individuals aged ≥50 years, referred to in this study as “older adults”.

## 2 METHODS

### 2.1 Study population and data sources

This study investigated the risk of SARS‐CoV‐2 infection and of severe, critical, or fatal COVID‐19 in the population of Qatar ≥50 years of age between February 5, 2020, the earliest record for a SARS‐CoV‐2 test in the country, and June 15, 2023. It analyzed the national, federated databases for COVID‐19 laboratory testing, vaccination, hospitalization, and death, retrieved from the integrated, nationwide, digital‐health information platform (Section S1 in Supplementary Appendix).

The databases contain SARS‐CoV‐2‐related data with no missing information since the pandemic's onset, including all polymerase chain reaction (PCR) tests regardless of location or facility and, from January 5, 2022, all medically supervised rapid antigen tests (Section S1). SARS‐CoV‐2 testing was, up to October 31, 2022, widely performed in Qatar with nearly 5% of the population tested every week, mostly for routine reasons, such as for screening or travel‐related purposes.^14^, ^15^ Although testing was scaled down as of November 1, 2022, close to 1% of the population were tested every week up to the end of the study. Most infections were diagnosed through testing for routine reasons rather than testing because of symptoms (Sections S1 and S2).^14^, ^15^

The national mortality database was used to obtain data on all‐cause mortality, including deaths occurring at healthcare facilities and elsewhere.^16^, ^17^ Qatar launched its COVID‐19 vaccination program using mRNA vaccines in December of 2020, prioritizing individuals by age.^18^ COVID‐19 vaccination was provided free of charge regardless of citizenship or residency status. Demographic information, such as sex, age, and nationality were extracted as registered in the national health registry. Qatar has remarkably diverse demographics, with 89% of its residents being expatriates hailing from over 150 countries.^19^ Detailed descriptions of Qatar's population and national databases have been previously reported.^14^, ^15^, ^16^, ^17^, ^19^, ^20^, ^21^

### 2.2 Study design and follow‐up

We conducted a retrospective cohort study to investigate incidence and risk factors for SARS‐CoV‐2 primary infection and associated severe, critical, or fatal COVID‐19 in the national cohort of individuals ≥50 years of age. All individuals ≥50 years of age at the start of the study on February 5, 2020 were eligible for inclusion. Individuals were followed until their first documented SARS‐CoV‐2 infection (regardless of symptoms), or death, or administrative end of follow‐up (June 15, 2023).

Incidence of documented infection was defined as the first documented PCR‐positive or rapid‐antigen‐positive test after the start of follow‐up, regardless of symptoms. Infection severity was classified following World Health Organization guidelines for COVID‐19 case severity (acute‐care hospitalizations),^12^ criticality (intensive‐care‐unit hospitalizations),^12^ and fatality^13^ (Section S3). Patients who progressed to severe, critical, or fatal COVID‐19 after a documented infection were classified based on their worst assessment outcome related to that infection, starting with COVID‐19 death,^13^ followed by critical disease,^12^ and then severe disease^12^ (Section S3). The date of incidence of severe COVID‐19 outcomes was set as the day of the SARS‐CoV‐2‐positive test that documented the infection that progressed into severe forms of COVID‐19.

### 2.3 Oversight

The institutional review boards at Hamad Medical Corporation and Weill Cornell Medicine–Qatar approved this retrospective study with a waiver of informed consent. The study was reported according to the Strengthening the Reporting of Observational Studies in Epidemiology (STROBE) guidelines (Table S1).

### 2.4 Statistical analysis

The cohort was described using frequency distributions and measures of central tendency. Cumulative incidence of documented infection, defined as the proportion of individuals at risk, whose primary endpoint during follow‐up was a documented infection (or a severe, critical, or fatal COVID‐19), was estimated using the Kaplan–Meier estimator method. Incidence rate of infection (and of severe COVID‐19 outcomes), defined as the number of identified infections (or severe COVID‐19 outcomes) divided by the number of person‐years contributed by all individuals in the cohort, was estimated, with the corresponding 95% confidence interval (CI), using a Poisson log‐likelihood regression model with the Stata 18.0 *stptime* command.

Hazard ratios (HRs), comparing incidence of infection across 5‐year age groups, number of coexisting conditions (0, 1, 2, 3, 4, 5, or ≥6 coexisting conditions), sex, and vaccination dose status, along with the corresponding 95% CIs, were calculated using multivariable Cox regression models. HRs were further adjusted for 10 nationality groups and testing rate. Low, intermediate, and high testers were defined as individuals with <1, 1–3, and ≥4 tests per person‐year, respectively, during follow‐up. To account for changes in vaccination status over time, vaccination was included in all models as a time‐varying covariate. Standard errors were adjusted for clustering effects. Interactions were not investigated.

The study analyzed infections and severe COVID‐19 outcomes that occurred in Qatar. However, certain outcomes might have occurred outside Qatar when expatriates were traveling abroad or permanently left the country after the follow‐up started. Consequently, an additional complete set of study results was generated through a sensitivity analysis restricted only to Qataris, thereby excluding all expatriates. The objective was to assess whether the travel or departure of expatriates from the country could have introduced bias that influenced the study's findings. Statistical analyses were performed using Stata/SE version 18.0 (Stata Corporation, College Station, TX, USA).

## 3 RESULTS

### 3.1 Study population

**FIGURE 1:**
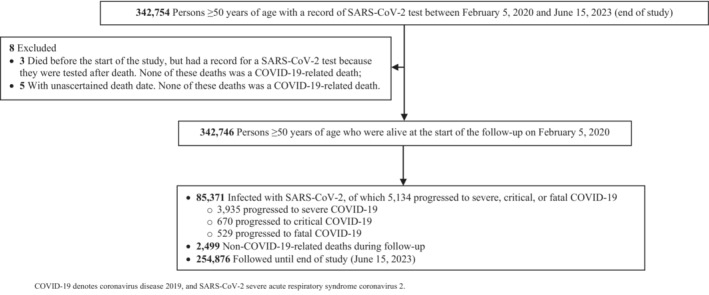
Flowchart illustrating the selection process of the study population.

Figure 1 depicts the procedure for selecting the study population. Table 1 provides an overview of the baseline characteristics of the study cohort. The national cohort comprised 342 746 individuals aged ≥50 years. Approximately half of the study cohort fell within the 50–54 age range, and roughly two‐thirds were males. The national backgrounds of the cohort were varied, with the largest group being Indians, followed by Qataris. More than a third of participants had documented records of at least one coexisting medical condition, and the majority of individuals received at least one vaccine dose during the follow‐up period. The baseline characteristics of the study cohort restricted to only Qataris are shown in Table S2.

**TABLE 1:** Baseline characteristics of the study cohort.

Characteristics of study cohort	N (%)
	N = 342 746
Median age at the start of follow‐up (IQR)—years	55.7 (52.2–60.9)
Age—years	
50–54	156 631 (45.7)
55–59	88 371 (25.8)
60–64	50 243 (14.7)
65–69	17 590 (5.1)
70–74	15 969 (4.7)
75–79	7668 (2.2)
80+	6274 (1.8)
Sex	
Male	232 537 (67.8)
Female	110 209 (32.2)
Nationality^a^	
Bangladeshi	16 374 (4.8)
Egyptian	16 579 (4.8)
Filipino	17 496 (5.1)
Indian	94 013 (27.4)
Nepalese	6659 (1.9)
Pakistani	18 026 (5.3)
Qatari	42 455 (12.4)
Sri Lankan	9535 (2.8)
Sudanese	11 095 (3.2)
Other nationalities^b^	110 514 (32.2)
Number of coexisting medical conditions	
None	220 923 (64.5)
1	32 216 (9.4)
2	29 181 (8.5)
3	20 677 (6.0)
4	16 301 (4.8)
5	10 925 (3.2)
6+	12 523 (3.7)
Vaccine dose^c^	
0	158 228 (46.2)
1	6383 (1.9)
2	89 744 (26.2)
3	80 221 (23.4)
4	8153 (2.4)
5	17 (<0.01)
Testing frequency^d^	
Low	191 136 (55.8)
Intermediate	126 446 (36.9)
High	25 164 (7.3)

IQR denotes interquartile range.

^a^ Nationalities were chosen to represent the most populous groups in Qatar.

^b^ These comprise up to 177 other nationalities.

^c^ Ascertained at time of censoring.

^d^ Low, intermediate, and high testers were defined as individuals with <1, 1–3, and ≥4 tests per person‐year, respectively, during follow‐up.

### 3.2 Cohort follow‐up

**FIGURE 2:**
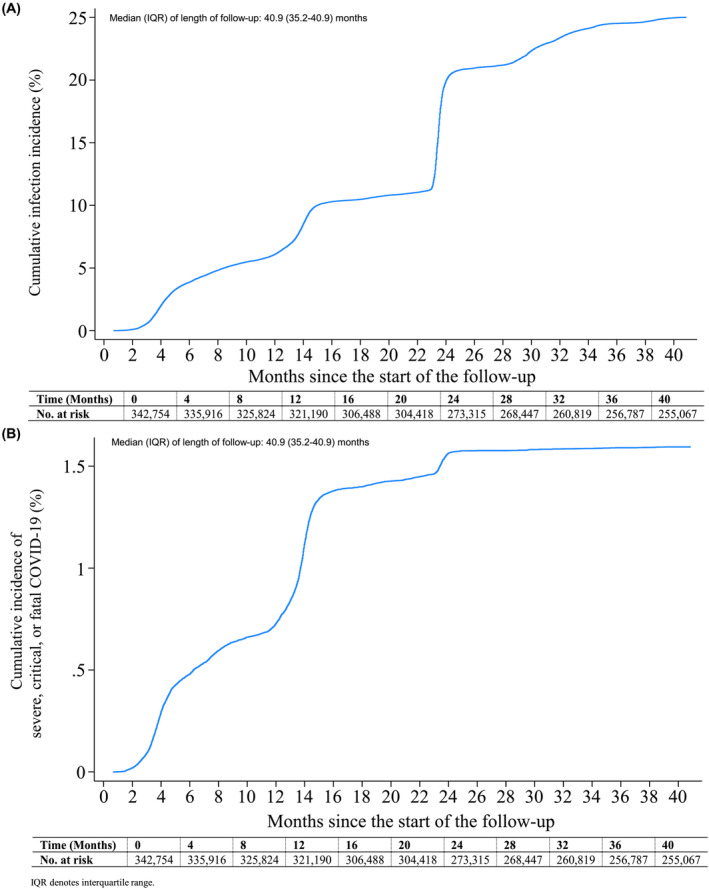
Cumulative incidence of (A) SARS‐CoV‐2 infection and (B) severe, critical, or fatal COVID‐19 disease over the duration of the study.

The majority of the cohort was followed until the conclusion of the study, covering a median follow‐up duration of 40.9 months, with an interquartile range (IQR) of 35.2–40.9 months (Figure 2). Throughout this follow‐up duration, a total of 85 371 primary SARS‐CoV‐2 infections were recorded. Among these cases, 3935 infections progressed to severe COVID‐19, 670 to critical COVID‐19, and 529 resulted in fatal COVID‐19 outcomes. For the subcohort of only Qataris, a total of 22 203 primary SARS‐CoV‐2 infections were recorded. Among these cases, 719 infections progressed to severe COVID‐19, 136 to critical COVID‐19, and 125 resulted in fatal COVID‐19 outcomes.

### 3.3 Incidence of infection

The cumulative incidence of documented infection reached 25.01% (95% CI: 24.86–25.15%) by the end of the study at the 40.9‐month mark since the start of follow‐up (Figure 2A). In addition to these documented cases of infection, many other infections may have never been documented.^22^, ^23^ Before the onset of the omicron wave on December 19, 2021,^20^, ^24^ the cumulative incidence increased rapidly during the ancestral virus,^19^ alpha,^25^ and beta^26^ waves (note details on waves and variants in Section S4 and Figure S1). By the end of the pre‐omicron phase, the cumulative incidence reached 11.39% (95% CI: 11.29–11.50%) at the 23‐month mark of follow‐up.

The onset of the omicron wave led to a very rapid increase in the cumulative incidence of infection, reaching 19.93% (95% CI: 19.80–20.07%) within only one month (at the 24‐month mark). Cumulative incidence continued to increase thereafter, but at a slower pace. The incidence rate of infection throughout the duration of follow‐up was estimated at 86.14 (95% CI: 85.57–86.72) per 1000 person‐years. Similar results were found in the sensitivity analysis restricted only to Qataris (Figure S2A).

### 3.4 Incidence of severe, critical, or fatal COVID‐19

The cumulative incidence of severe, critical, or fatal COVID‐19 reached 1.59% (95% CI: 1.55–1.64%) by the end of the study at the 40.9‐month mark since the start of follow‐up (Figure 2B). Before the onset of the omicron wave on December 19, 2021,^20^, ^24^ the cumulative incidence exhibited its most rapid increase during the combined alpha^25^ and beta^26^ waves that sequentially followed each other (Section S4 and Figure S1). As the pre‐omicron phase concluded, the cumulative incidence reached 1.46% (95% CI: 1.42–1.50%) at the 23‐month mark of follow‐up.

Despite the onset of the omicron wave leading to a very large surge in the incidence of infection (Figure 2A), the rise in the incidence of severe, critical, or fatal COVID‐19 was comparatively modest. It only reached 1.56% (95% CI: 1.52–1.61%) at the 24‐month mark. The cumulative incidence of severe, critical, or fatal COVID‐19 remained flat, with hardly any further increase after the conclusion of the first omicron wave. The incidence rate of severe, critical, or fatal COVID‐19 throughout the duration of follow‐up was estimated at 5.18 (95% CI: 5.04–5.32) per 1000 person‐years. Similar results were found in the sensitivity analysis restricted only to Qataris (Figure S2B).

### 3.5 Effects of age, coexisting conditions, sex, and vaccination on infection

**FIGURE 3:**
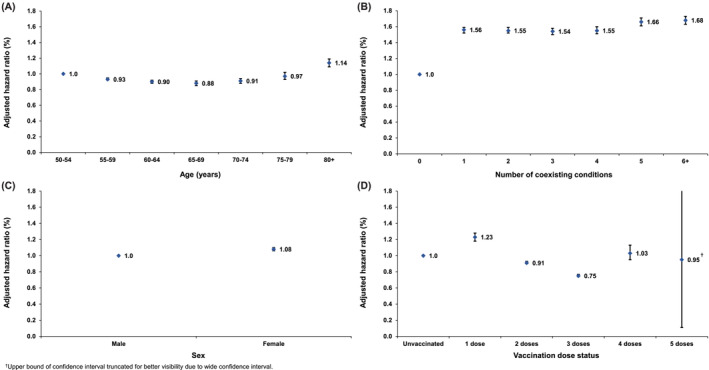
Adjusted hazard ratios against SARS‐CoV‐2 infection across (A) age, (B) number of coexisting conditions, (C) sex, and (D) vaccination dose status.

Figure 3 and Table 2 present the adjusted HRs (AHRs) for SARS‐CoV‐2 infection across 5‐year age groups, number of coexisting conditions, sex, and vaccination dose status. Only minor differences were noted in the infection incidence by age and sex. The presence of one or more coexisting conditions was associated with significantly higher incidence of infection when compared with individuals without a record of coexisting conditions. Primary‐series vaccination and first booster vaccination were both associated with a reduced incidence of infection in comparison with the unvaccinated group, with AHRs of 0.91 (95% CI: 0.90–0.93) and 0.75 (95% CI: 0.74–0.77), respectively. The sensitivity analysis restricted only to Qataris demonstrated largely similar findings (Figure S3 and Table S3).

**TABLE 2:** Adjusted hazard ratios against SARS‐CoV‐2 infection and against severe, critical, or fatal COVID‐19 disease across age, number of coexisting conditions, sex, and vaccination dose status.

Characteristics	Adjusted hazard ratio^a^ (95% CI^c^)	
	Against SARS‐CoV‐2 infection	Against severe, critical, or fatal COVID‐19^b^
Age—years		
50‐54	1.00	1.00
55–59	0.93 (0.92–0.95)	1.16 (1.08–1.25)
60–64	0.90 (0.88–0.92)	1.34 (1.24–1.47)
65–69	0.88 (0.85–0.91)	1.53 (1.36–1.72)
70–74	0.91 (0.88–0.94)	1.99 (1.78–2.22)
75–79	0.97 (0.93–1.02)	2.49 (2.17–2.84)
80+	1.14 (1.09–1.19)	3.90 (3.46–4.39)
Number of coexisting conditions		
None	1.00	1.00
1	1.56 (1.52–1.59)	6.30 (5.73–6.93)
2	1.55 (1.52–1.59)	7.41 (6.74–8.15)
3	1.54 (1.50–1.58)	8.65 (7.80–9.58)
4	1.55 (1.51–1.60)	8.82 (7.90–9.85)
5	1.66 (1.61–1.71)	10.69 (9.49–12.05)
6+	1.68 (1.63–1.73)	13.06 (11.69–14.59)
Sex		
Male	1.00	1.00
Female	1.08 (1.06–1.10)	0.54 (0.50–0.57)
Vaccination dose status		
0	1.00	1.00
1	1.23 (1.18–1.28)	0.86 (0.77–0.97)
2	0.91 (0.90–0.93)	0.15 (0.13–0.17)
3	0.75 (0.74–0.77)	0.11 (0.08–0.14)
4	1.03 (0.95–1.13)	0.00 (0.00–0.04)^c^
5	0.95 (0.11–7.97)	0.00 (0.00–17.44)^c^

CI denotes confidence interval COVID‐19, coronavirus disease 2019, and SARS‐CoV‐2, severe acute respiratory syndrome coronavirus 2.

^a^ Adjusted for 5‐year age groups, number of coexisting conditions, sex, vaccination dose status, 10 nationality groups, and testing rate.

^b^ Severity,^12^ criticality,^12^ and fatality^13^ were defined according to the World Health Organization guidelines.

^c^ CI for the adjusted hazard ratio could not be estimated because of zero events among those vaccinated and was approximated by the CI for the odds ratio obtained using the Cornfield method.

### 3.6 Effects of age, coexisting conditions, sex, and vaccination on severe, critical, or fatal COVID‐19

**FIGURE 4:**
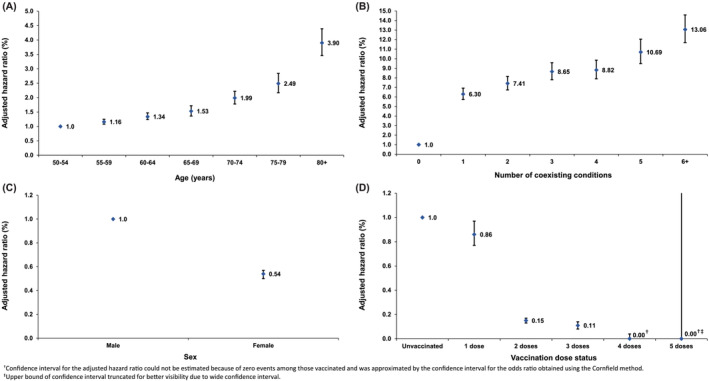
Adjusted hazard ratios against severe, critical, or fatal COVID‐19 disease across (A) age, (B) number of coexisting conditions, (C) sex, and (D) vaccination dose status.

Figure 4 and Table 2 present the AHRs for severe, critical, or fatal COVID‐19 across 5‐year age groups, number of coexisting conditions, sex, and vaccination dose status. Age and number of existing conditions exhibited strong associations with the incidence of severe, critical, or fatal COVID‐19. The incidence of severe, critical, or fatal COVID‐19 demonstrated an exponential increase with age and a relatively linear relationship with the number of coexisting conditions.

Notably, women had a substantially lower incidence of severe, critical, or fatal COVID‐19 compared with men. Number of vaccine doses showed a significant association with a reduced incidence of severe, critical, or fatal COVID‐19. In comparison with the unvaccinated group, the AHR was 0.86 (95% CI: 0.7–0.97) for individuals who received only one dose, 0.15 (95% CI: 0.13–0.17) for those who received primary‐series vaccination, and 0.11 (95% CI: 0.08–0.14) for those who received a first booster vaccination. The sensitivity analysis restricted only to Qataris demonstrated largely similar findings (Figure S4 and Table S3).

## 4 DISCUSSION

Incidence of severe COVID‐19 among older adults varied throughout the pandemic reflecting differences in infection incidence (Figure S1), severity of variants,^25^, ^27^, ^28^, ^29^, ^30^, ^31^, ^32^ and level of population immunity.^33^, ^34^, ^35^ Overall, nearly 2% of the population older than 50 years of age in Qatar developed severe forms of COVID‐19 over a duration of three and half years of follow‐up. Nearly all severe COVID‐19 cases occurred before the conclusion of the first omicron wave. After this wave, incidence of severe COVID‐19 was rare among older adults, possibly because of rapid accrual of natural immunity during this first omicron wave.^36^ Vaccinations and advancements in case management also contributed to reducing severity and fatality over time, but only gradually.^15^, ^20^, ^37^

Severe forms of COVID‐19 were strongly associated with age and number of coexisting conditions among older individuals. Even minor differences in age led to an exponential increase in the risk of severe COVID‐19. Severe COVID‐19 increased proportionally with the number of existing conditions. These findings align with the widely observed global patterns, highlighting the roles of age and underlying health conditions in infection progression to severe COVID‐19 among older individuals.^1^, ^2^ Findings are also consistent with accelerated deaths among older adults and those with coexisting conditions being a major driver of COVID‐19 mortality in Qatar.^17^ These findings may also suggest that, specifically among older individuals, the effect of age on severe COVID‐19 could outweigh the effect of coexisting conditions. The findings also support the concept of COVID‐19 being an emerging typical aging disease.^9^

Women were considerably less likely to progress to severe COVID‐19 than men, even after adjustment for the effects of age and coexisting conditions. This is consistent with global evidence and may reflect biological differences between men and women that extend beyond underlying health conditions.^38^, ^39^, ^40^, ^41^, ^42^ This finding may also reflect under‐ascertainment of coexisting conditions among men, considering that women have a higher tendency to seek preventive care and to utilize health services than men.^43^

As expected, vaccination provided strong protection against severe COVID‐19 in older adults, retaining its effectiveness over a span of approximately three years of follow‐up. The observed pattern indicated a dose–response relationship where a higher number of vaccine doses elicited greater protection. While affirming the protective effects of vaccination within this population^15^, ^20^, ^33^, ^44^ and corroborating global literature,^45^, ^46^ this finding further emphasizes the criticality of vaccination for older persons.

Although some disparities in infection risk based on age, sex, and the number of coexisting conditions were observed, most of these differences were modest and could pose challenges for interpretation. These differences could stem from various factors, including variations in social networks, which might differ significantly with age among older adults because of retirement or reduced mobility and functionality, as well as frailty. These differences may also reflect variations in health‐seeking behaviors, beyond the controlled factor of testing frequency considered in this analysis.

Despite a protective effect for vaccination against infection, the magnitude of protection was notably much smaller (over the entire time of follow‐up) compared with that against severe COVID‐19. This divergence can be attributed to the rapid waning of vaccine protection against infection, which lasts for only a few months after the last dose,^15^, ^21^, ^33^, ^44^, ^45^, ^46^, ^47^ and thus did not extend over the three years of follow‐up in this study.

This study has limitations. The study analyzed infection and COVID‐19 outcomes that occurred in Qatar, but some outcomes may have occurred outside Qatar, while expatriates were traveling abroad, or if they have left Qatar permanently because of end of employment or other reasons after initiation of follow‐up. Travel data were not available to factor in our analysis. With the restrictions on travel and international recruitment during the pandemic, such movements in and out of the country were probably limited in scale in the early phases of the pandemic but increased with time as restrictions were gradually lifted. Mid to end of 2022 in particular, was a time during which such migration movements have increased substantially as some expatriates may have left Qatar with the end of World Cup 2022 projects. Such migration movements could potentially introduce bias that affect the results because of differential loss of follow‐up for expatriates compared with Qataris. To investigate effect of this bias, we generated a complete set of study results for only Qataris, and these confirmed similar findings, suggesting that this bias may not have appreciably affected the findings. There were only small differences, and these affected only the effect sizes and not direction of effects between the results with and without inclusion of expatriates.

As an observational study, unmeasured or uncontrolled confounding cannot be excluded. While we adjusted for several factors in our analyses, other factors were not available to adjust for. The study approach was to investigate broad effects over the entire duration of follow‐up, but the effect of confounding factors may have varied with calendar time, thereby potentially introducing bias. For example, having one dose of vaccination was associated with slightly higher risk of infection than no vaccination. However, this effect is likely to reflect uncontrolled bias arising from the temporal effect of most individuals receiving their first dose during a time of high infection incidence, specifically during the alpha and beta waves.^25^, ^26^

The study analyzed documented infections, but some infections may have gone undocumented, particularly after the major reduction in testing that occurred starting from November 1, 2022.^32^ Earlier serological surveys and other analyses conducted in Qatar and elsewhere indicated that a considerable proportion of infections are undocumented.^2^, ^23^, ^48^, ^49^, ^50^, ^51^, ^52^ With the absence of recent serological surveys in Qatar, estimating the current or recent infection detection rate is challenging. Nevertheless, mathematical modeling analyses for Qatar and their updates suggest that at least 50% of infections have never been documented.^22^, ^23^

COVID‐19 severity in Qatar's population was relatively low, but this finding may not be generalizable to other countries. Qatar, with its high human development index,^53^ well‐resourced healthcare system,^54^ and low threshold for hospital admission for older adults presenting with COVID‐19,^16^, ^19^ has witnessed lower severity and fatality rates of COVID‐19 than other countries.^2^, ^16^, ^17^

While these limitations may affect some of the results relating to infection, they are less likely to affect the results relating to severe COVID‐19, as severe COVID‐19 cases are not likely to be undocumented and COVID‐19 severity was assessed using the standardized WHO classification of COVID‐19 severity,^12^, ^13^ which was applied by trained medical personnel that evaluated the severity using a national protocol applied to every hospitalized COVID‐19 patient.^2^, ^15^ COVID‐19‐associated hospitalizations were not used as a proxy for COVID‐19 severity, as these have limitations in accurately capturing the true severity of COVID‐19.^55^, ^56^

In conclusion, incidence of severe COVID‐19 among older adults in Qatar displayed a dynamic pattern throughout the pandemic, influenced by infection rates, variant severity, and population immunity. The vast majority of severe cases occurred prior to the conclusion of the first omicron wave, with a major subsequent decline in incidence because of the rapid accumulation of natural immunity during the first omicron wave, as well as gradual improvements in vaccine coverage and case management. Age and number of coexisting conditions were identified as strong determinants of severe COVID‐19 risk among older adults, with even minor differences having a significant impact on progression from infection to severe COVID‐19. Despite the rapid waning of vaccine protection against infection, vaccination emerged as a critical protective measure against severe outcomes, maintaining its effectiveness over three years and demonstrating a clear dose–response relationship with the number of doses administered. These findings emphasize the importance of booster vaccination in mitigating the impact of predisposing factors for severe COVID‐19 in older adults.

## AUTHOR CONTRIBUTIONS

**Mai A. Mahmoud:** Conceptualization; data curation; methodology; writing—original draft; writing—review and editing. **Houssein H. Ayoub:** Data curation; writing—review and editing. **Peter Coyle:** Data curation; investigation; writing—review and editing. **Patrick Tang:** Data curation; investigation; writing—review and editing. **Mohammad R. Hasan:** Data curation; investigation; writing—review and editing. **Hadi M. Yassine:** Data curation; investigation; writing—review and editing. **Asmaa A. Al Thani:** Data curation; investigation; writing—review and editing. **Zaina Al‐Kanaani:** Data curation; writing—review and editing. **Einas Al‐Kuwari:** Data curation; writing—review and editing. **Andrew Jeremijenko:** Data curation; writing—review and editing. **Anvar Hassan Kaleeckal:** Data curation; writing—review and editing. **Ali Nizar Latif:** Data curation; writing—review and editing. **Riyazuddin Mohammad Shaik:** Data curation; writing—review and editing. **Hanan F. Abdul‐Rahim:** Data curation; writing—review and editing. **Gheyath K. Nasrallah:** Data curation; writing—review and editing. **Mohamed Ghaith Al‐Kuwari:** Data curation; writing—review and editing. **Adeel A. Butt:** Data curation; writing—review and editing. **Hamad Eid Al‐Romaihi:** Data curation; writing—review and editing. **Mohamed H. Al‐Thani:** Data curation; writing—review and editing. **Abdullatif Al‐Khal:** Data curation; writing—review and editing. **Roberto Bertollini:** Data curation; writing—review and editing. **Laith J. Abu‐Raddad:** Conceptualization; data curation; methodology; project administration; supervision; writing—original draft; writing—review and editing. **Hiam Chemaitelly:** Conceptualization; data curation; formal analysis; methodology; writing—original draft; writing—review and editing.

## CONFLICT OF INTEREST STATEMENT

Dr. Butt has received institutional grant funding from Gilead Sciences unrelated to the work presented in this paper. Otherwise, we declare no conflict of interest.

### PEER REVIEW

The peer review history for this article is available at https://www.webofscience.com/api/gateway/wos/peer-review/10.1111/irv.13224.

## ETHICS STATEMENT

Hamad Medical Corporation and Weill Cornell Medicine‐Qatar Institutional Review Boards approved this retrospective study with a waiver of informed consent.

## Supporting information


**TABLE S1.** STROBE checklist for cohort studies.
**Table S2.** Baseline characteristics of Qatari participants.
**Figure S1.** Daily count of newly diagnosed SARS‐CoV‐2 infections between February 5, 2020 and June 15, 2023.
**Figure S2.** Cumulative incidence of A) SARS‐CoV‐2 infection and B) severe, critical, or fatal COVID‐19 disease among Qataris over the duration of the study.
**Figure S3.** Adjusted hazard ratios against SARS‐CoV‐2 infection among Qataris across A) age, B) number of coexisting conditions, C) sex, and D) vaccination dose status.
**Table S3.** Adjusted hazard ratios against SARS‐CoV‐2 infection and against severe, critical, or fatal COVID‐19 disease among Qataris across age, number of coexisting conditions, sex, and vaccination status.
**Figure S4.** Adjusted hazard ratios against severe, critical, or fatal COVID‐19 disease among Qataris across A) age, B) number of coexisting conditions, C) sex, and D) vaccination dose status.
**SECTION S1.** Study population and data sources.
**SECTION S2.** Laboratory methods and variant ascertainment
Real‐time reverse‐transcription polymerase chain reaction testing.
Rapid antigen testing
Classification of infections by variant type
**SECTION S3.** COVID‐19 severity, criticality, and fatality classification
Severe COVID‐19
Critical COVID‐19
Fatal COVID‐19
**SECTION S4.** Phases of the COVID‐19 pandemic
**SECTION S5.** Comorbidity classification

## Data Availability

The dataset of this study is a property of the Qatar Ministry of Public Health that was provided to the researchers through a restricted‐access agreement that prevents sharing the dataset with a third party or publicly. The data are available under restricted access for preservation of confidentiality of patient data. Access can be obtained through a direct application for data access to Her Excellency the Minister of Public Health (https://www.moph.gov.qa/english/OurServices/eservices/Pages/Governmental‐HealthCommunication‐Center.aspx). The raw data are protected and are not available due to data privacy laws. Aggregate data are available within the paper and its supplementary information.
