# Supplementary material for: SARS‐CoV‐2 infection and effects of age, sex, comorbidity, and vaccination among older individuals: A national cohort study
Source: Influenza Other Respir Viruses. 2023 Nov 21;17(11):e13224. doi: 10.1111/irv.13224 (PMC10663173; doi:10.1111/irv.13224)
Supplement: Supplementary file 1 — TABLE S1. STROBE checklist for cohort studies. Table S2. Baseline characteristics of Qatari participants. Figure S1. Daily count of newly diagnosed SARS‐CoV‐2 infections between February 5, 2020 and June 15, 2023. Figure S2. Cumulative incidence of A) SARS‐CoV‐2 infection and B) severe, critical, or fatal COVID‐19 disease among Qataris over the duration of the study. Figure S3. Adjusted hazard ratios against SARS‐CoV‐2 infection among Qataris across A) age, B) number of coexisting conditions, C) sex, and D) vaccination dose status. Table S3. Adjusted hazard ratios against SARS‐CoV‐2 infection and against severe, critical, or fatal COVID‐19 disease among Qataris across age, number of coexisting conditions, sex, and vaccination status. Figure S4. Adjusted hazard ratios against severe, critical, or fatal COVID‐19 disease among Qataris across A) age, B) number of coexisting conditions, C) sex, and D) vaccination dose status. SECTION S1. Study population and data sources. SECTION S2. Laboratory methods and variant ascertainment Real‐time reverse‐transcription polymerase chain reaction testing. Rapid antigen testing Classification of infections by variant type SECTION S3. COVID‐19 severity, criticality, and fatality classification Severe COVID‐19 Critical COVID‐19 Fatal COVID‐19 SECTION S4. Phases of the COVID‐19 pandemic SECTION S5. Comorbidity classification [file IRV-17-e13224-s001.docx]

**Supplementary Information**

**Table of Contents**

[SECTION S1. Study population and data sources 2](#_Toc148891196)

[SECTION S2. Laboratory methods and variant ascertainment 5](#_Toc148891197)

[**Real-time reverse-transcription polymerase chain reaction testing** 5](#_Toc148891198)

[**Rapid antigen testing** 5](#_Toc148891199)

[**Classification of infections by variant type** 6](#_Toc148891200)

[SECTION S3. COVID-19 severity, criticality, and fatality classification 7](#_Toc148891201)

[**Severe COVID-19** 7](#_Toc148891202)

[**Critical COVID-19** 7](#_Toc148891203)

[**Fatal COVID-19** 8](#_Toc148891204)

[SECTION S4. Phases of the COVID-19 pandemic 9](#_Toc148891205)

[SECTION S5. Comorbidity classification 10](#_Toc148891206)

[TABLE S1. STROBE checklist for cohort studies. 11](#_Toc148891207)

[TABLE S2. Baseline characteristics of Qatari participants. 13](#_Toc148891208)

[FIGURE S1. Daily count of newly diagnosed SARS-CoV-2 infections between February 5, 2020 and June 15, 2023. 14](#_Toc148891209)

[FIGURE S2. Cumulative incidence of A) SARS-CoV-2 infection and B) severe, critical, or fatal COVID-19 disease among Qataris over the duration of the study. 15](#_Toc148891210)

[FIGURE S3. Adjusted hazard ratios against SARS-CoV-2 infection among Qataris across A) age, B) number of coexisting conditions, C) sex, and D) vaccination dose status. 16](#_Toc148891211)

[TABLE S3. Adjusted hazard ratios against SARS-CoV-2 infection and against severe, critical, or fatal COVID-19 disease among Qataris across age, number of coexisting conditions, sex, and vaccination status. 17](#_Toc148891212)

[FIGURE S4. Adjusted hazard ratios against severe, critical, or fatal COVID-19 disease among Qataris across A) age, B) number of coexisting conditions, C) sex, and D) vaccination dose status. 18](#_Toc148891213)

[REFERENCES 19](#_Toc148891214)

#

# **SECTION S1. Study population and data sources**

Qatar’s national and universal public healthcare system uses the Cerner-system advanced digital health platform to track all electronic health record encounters of each individual in the country, including all citizens and residents registered in the national and universal public healthcare system. Registration in the public healthcare system is mandatory for citizens and residents.

The databases analyzed in this study are data-extract downloads from the Cerner-system that have been implemented on a regular (twice weekly) schedule since onset of the pandemic by the Business Intelligence Unit at Hamad Medical Corporation. Hamad Medical Corporation is the national public healthcare provider in Qatar. At every download all tests, coronavirus disease 2019 (COVID-19) vaccinations, hospitalizations related to COVID-19, and all death records regardless of cause are provided to the authors through .csv files. These databases have been analyzed throughout the pandemic not only for study-related purposes, but also to provide policymakers with summary data and analytics to inform the national response.

Every health encounter in the Cerner-system is linked to a unique individual through the HMC Number that links all records for this individual at the national level. Databases were merged and analyzed using the HMC Number to link all records whether for testing, vaccinations, hospitalizations, and deaths. All deaths in Qatar are tracked by the public healthcare system. All COVID-19-related healthcare was provided only in the public healthcare system. No private entity was permitted to provide COVID-19-related hospitalization. COVID-19 vaccination was also provided only through the public healthcare system. These health records were tracked throughout the COVID-19 pandemic using the Cerner system. This system has been implemented in 2013, before the onset of the pandemic. Therefore, we had all health records related to this study for the full national cohort of Qataris throughout the pandemic. This allowed us to follow each person over time.

Demographic details for every HMC Number (individual) such as sex, age, and nationality are collected upon issuing of the universal health card, based on the Qatar Identity Card, which is a mandatory requirement by the Ministry of Interior to every citizen and resident in the country. Data extraction from the Qatar Identity Card to the digital health platform is performed electronically through scanning techniques.

All severe acute respiratory syndrome coronavirus 2 (SARS-CoV-2) testing in any facility in Qatar is tracked nationally in one database, the national testing database. This database covers all testing in all locations and facilities throughout the country, whether public or private. Every polymerase chain reaction (PCR) test and a proportion of the facility-based rapid antigen tests conducted in Qatar, regardless of location or setting, are classified on the basis of symptoms and the reason for testing (clinical symptoms, contact tracing, surveys or random testing campaigns, individual requests, routine healthcare testing, pre-travel, at port of entry, or other).

Before November 1, 2022, SARS-CoV-2 testing in Qatar was done at a mass scale where about 5% of the population were tested every week.^1^ Based on the distribution of the reason for testing up to November 1, 2022, most of the tests in Qatar were conducted for routine reasons, such as being travel-related, and about 75% of cases were diagnosed not because of appearance of symptoms, but because of routine testing.^1,2^

Starting from November 1, 2022, SARS-CoV-2 testing was substantially reduced, but still about 1% of the population are tested every week.^2^ All testing results in the national testing database during follow-up in the present study were factored in the analyses of this study.

The first large omicron wave that peaked in January of 2022 was massive and strained the testing capacity in the country.^1,3-5^ Accordingly, rapid antigen testing was introduced to relieve the pressure on PCR testing. Implementation of this change in testing policy occurred quickly precluding incorporation of reason for testing in a large proportion of the rapid antigen tests for several months. While the reason for testing is available for all PCR tests, it is not available for all rapid antigen tests. Availability of reason for testing for the rapid antigen tests also varied with time.

Rapid antigen test kits are available for purchase in pharmacies in Qatar, but outcome of home-based testing is not reported nor documented in the national databases. Since SARS-CoV-2-test outcomes were linked to specific public health measures, restrictions, and privileges, testing policy and guidelines stress facility-based testing as the core testing mechanism in the population. While facility-based testing is provided free of charge or at low subsidized costs, depending on the reason for testing, home-based rapid antigen testing is de-emphasized and not supported as part of national policy.

Further descriptions of the study population and the national databases were reported previously.^1,2,5-10^

# **SECTION S2. Laboratory methods and variant ascertainment**

## **Real-time reverse-transcription polymerase chain reaction testing**

Nasopharyngeal and/or oropharyngeal swabs were collected for polymerase chain reaction (PCR) testing and placed in Universal Transport Medium (UTM). Aliquots of UTM were: 1) extracted on KingFisher Flex (Thermo Fisher Scientific, USA), MGISP-960 (MGI, China), or ExiPrep 96 Lite (Bioneer, South Korea) followed by testing with real-time reverse-transcription PCR (RT-qPCR) using TaqPath COVID-19 Combo Kits (Thermo Fisher Scientific, USA) on an ABI 7500 FAST (Thermo Fisher Scientific, USA); 2) tested directly on the Cepheid GeneXpert system using the Xpert Xpress SARS-CoV-2 (Cepheid, USA); or 3) loaded directly into a Roche cobas 6800 system and assayed with the cobas SARS-CoV-2 Test (Roche, Switzerland). The first assay targets the viral S, N, and ORF1ab gene regions. The second targets the viral N and E-gene regions, and the third targets the ORF1ab and E-gene regions.

All PCR testing was conducted at the Hamad Medical Corporation Central Laboratory or Sidra Medicine Laboratory, following standardized protocols.

## **Rapid antigen testing**

Severe acute respiratory syndrome coronavirus 2 (SARS-CoV-2) antigen tests were performed on nasopharyngeal swabs using one of the following lateral flow antigen tests: Panbio COVID-19 Ag Rapid Test Device (Abbott, USA); SARS-CoV-2 Rapid Antigen Test (Roche, Switzerland); Standard Q COVID-19 Antigen Test (SD Biosensor, Korea); or CareStart COVID-19 Antigen Test (Access Bio, USA). All antigen tests were performed point-of-care according to each manufacturer’s instructions at public or private hospitals and clinics throughout Qatar with prior authorization and training by the Ministry of Public Health (MOPH). Antigen test results were electronically reported to the MOPH in real time using the Antigen Test Management System which is integrated with the national Coronavirus Disease 2019 (COVID-19) database.

## **Classification of infections by variant type**

Surveillance for SARS-CoV-2 variants in Qatar is based on viral genome sequencing and multiplex RT-qPCR variant screening^9^ of random positive clinical samples,^11-16^ complemented by deep sequencing of wastewater samples.^2,14,17^ Further details on the viral genome sequencing and multiplex RT-qPCR variant screening throughout the SARS-CoV-2 waves in Qatar can be found in previous publications.^1,2,4,8,12-16,18-22^

# **SECTION S3. COVID-19 severity, criticality, and fatality classification**

Classification of Coronavirus Disease 2019 (COVID-19) case severity (acute-care hospitalizations),^23^ criticality (intensive-care-unit hospitalizations),^24^ and fatality^24^ followed World Health Organization (WHO) guidelines. Assessments were made by trained medical personnel independent of study investigators and using individual chart reviews, as part of a national protocol applied to every hospitalized COVID-19 patient. Each hospitalized COVID-19 patient underwent an infection severity assessment every three days until discharge or death.

## **Severe COVID-19**

Severe COVID-19 disease was defined per WHO classification as a SARS-CoV-2 infected person with “oxygen saturation of <90% on room air, and/or respiratory rate of >30 breaths/minute in adults and children >5 years old (or ≥60 breaths/minute in children <2 months old or ≥50 breaths/minute in children 2-11 months old or ≥40 breaths/minute in children 1–5 years old), and/or signs of severe respiratory distress (accessory muscle use and inability to complete full sentences, and, in children, very severe chest wall indrawing, grunting, central cyanosis, or presence of any other general danger signs)”.^25^ Detailed WHO criteria for classifying Severe acute respiratory syndrome coronavirus 2 (SARS-CoV-2) infection severity can be found in the WHO technical report.^24^

## **Critical COVID-19**

Critical COVID-19 disease was defined per WHO classification as a SARS-CoV-2 infected person with “acute respiratory distress syndrome, sepsis, septic shock, or other conditions that would normally require the provision of life sustaining therapies such as mechanical ventilation (invasive or non-invasive) or vasopressor therapy”.^24^ Detailed WHO criteria for classifying SARS-CoV-2 infection criticality can be found in the WHO technical report.^24^

## **Fatal COVID-19**

COVID-19 death was defined per WHO classification as “a death resulting from a clinically compatible illness, in a probable or confirmed COVID-19 case, unless there is a clear alternative cause of death that cannot be related to COVID-19 disease (e.g. trauma). There should be no period of complete recovery from COVID-19 between illness and death. A death due to COVID-19 may not be attributed to another disease (e.g. cancer) and should be counted independently of preexisting conditions that are suspected of triggering a severe course of COVID-19”. Detailed WHO criteria for classifying COVID-19 death can be found in the WHO technical report.^24^

# **SECTION S4. Phases of the COVID-19 pandemic**

The pandemic was categorized into distinct phases based on the level of SARS-CoV-2 incidence and the predominant variant. These phases included the ancestral virus wave (February 28, 2020 - July 31, 2020),^25^ a prolonged low incidence phase with the ancestral virus (August 1, 2020 - January 17, 2021),^2,7^ the alpha wave (January 18, 2021 - March 7, 2021),^26^ the beta wave (March 8, 2021 - May 31, 2021),^27^ a prolonged low incidence delta phase (June 1, 2021 - December 18, 2021),^19,28^ the first (BA.1 & BA.2) omicron wave (December 19, 2021 - February 28, 2022),^29^ the omicron BA.4 & BA.5 wave (March 1, 2022 - September 9, 2022),^6^ and the omicron BA.2.75 & XBB waves (September 10, 2022 – June 15, 2023).^22^

# **SECTION S5. Comorbidity classification**

Comorbidities were ascertained and classified based on the ICD-10 codes as recorded in the electronic health record encounters of each individual in the Cerner-system national database that includes all citizens and residents registered in the national and universal public healthcare system. The public healthcare system provides healthcare to the entire resident population of Qatar free of charge or at heavily subsidized costs, including prescription drugs.

All encounters for each individual were analyzed to determine the comorbidity classification for that individual, as part of a recent national analysis to assess healthcare needs and resource allocation. The Cerner-system national database includes encounters starting from 2013, after this system was launched in Qatar. As long as each individual had at least one encounter with a specific comorbidity diagnosis since 2013, this person was classified with this comorbidity.

Individuals who have comorbidities but never sought care in the public healthcare system, or seek care exclusively in private healthcare facilities, were classified as individuals with no comorbidity due to absence of recorded encounters for them.

#

# **TABLE S1.** STROBE checklist for cohort studies.

|  | Item No | Recommendation | Main Text page |
| --- | --- | --- | --- |
| **Title and abstract** | 1 | (*a*) Indicate the study’s design with a commonly used term in the title or the abstract | Title and Abstract |
|  |  | (*b*) Provide in the abstract an informative and balanced summary of what was done and what was found |  |
| Introduction | | | |
| Background/rationale | 2 | Explain the scientific background and rationale for the investigation being reported | Introduction |
| Objectives | 3 | State specific objectives, including any prespecified hypotheses | Introduction |
| Methods | | | |
| Study design | 4 | Present key elements of study design early in the paper | Methods (‘Study design and follow-up’) |
| Setting | 5 | Describe the setting, locations, and relevant dates, including periods of recruitment, exposure, follow-up, and data collection | Methods (‘Study population and data sources’ & ‘Study design and follow-up’) & Sections S1-S5 in Supplementary Information |
| Participants | 6 | (*a*) Give the eligibility criteria, and the sources and methods of selection of participants. Describe methods of follow-up | Methods (‘Study population and data sources’ & ‘Study design and follow-up’) & Section S1 in Supplementary Information |
|  |  | (*b*) For matched studies, give matching criteria and number of exposed and unexposed |  |
| Variables | 7 | Clearly define all outcomes, exposures, predictors, potential confounders, and effect modifiers. Give diagnostic criteria, if applicable | Methods (‘Study design and follow-up’ & ‘Statistical analysis’), & Sections S1-S5 in Supplementary Information |
| Data sources/ measurement | 8* | For each variable of interest, give sources of data and details of methods of assessment (measurement). Describe comparability of assessment methods if there is more than one group | Methods, Table 1, & Sections S1-S5 & Table S2 in Supplementary Information |
| Bias | 9 | Describe any efforts to address potential sources of bias | Methods (‘Statistical analysis’) |
| Study size | 10 | Explain how the study size was arrived at | Figure 1 |
| Quantitative variables | 11 | Explain how quantitative variables were handled in the analyses. If applicable, describe which groupings were chosen and why | Methods (‘Statistical analysis’), Table 1, & Table S2 in Supplementary Information |
| Statistical methods | 12 | (*a*) Describe all statistical methods, including those used to control for confounding | Methods (‘Statistical analysis’) |
|  |  | (*b*) Describe any methods used to examine subgroups and interactions | Methods (‘Statistical analysis’) |
|  |  | (*c*) Explain how missing data were addressed | Not applicable, see Methods (‘Study population and data sources’) & Section S1 in Supplementary Information |
|  |  | (*d*) If applicable, explain how loss to follow-up was addressed | Not applicable, see Methods (‘Study population and data sources’) & Section S1 in Supplementary Information |
|  |  | (*e*) Describe any sensitivity analyses | Methods (‘Statistical analysis’) |
| Results | | |  |
| Participants | 13* | (a) Report numbers of individuals at each stage of study—eg numbers potentially eligible, examined for eligibility, confirmed eligible, included in the study, completing follow-up, and analysed | Results (‘Study population’), Figure 1, Table 1, & Table S2 in Supplementary Information |
|  |  | (b) Give reasons for non-participation at each stage |  |
|  |  | (c) Consider use of a flow diagram |  |
| Descriptive data | 14 | (a) Give characteristics of study participants (eg demographic, clinical, social) and information on exposures and potential confounders | Results (‘Study population’), Table 1, & Table S2 in Supplementary Information |
|  |  | (b) Indicate number of participants with missing data for each variable of interest | Not applicable, see Methods (‘Study population and data sources’) & Section S1 in Supplementary Information |
|  |  | (c) Summarise follow-up time (eg, average and total amount) | Results (‘Cohort follow-up’), Figure 2, & Figure S2 in Supplementary Information |
| Outcome data | 15 | Report numbers of outcome events or summary measures over time | Results (‘Cohort follow-up’, ‘Incidence of infection’, & ‘Incidence of severe, critical, or fatal COVID-19’), Figures 1-2, & Figure S2 in Supplementary Information |
| Main results | 16 | (a) Give unadjusted estimates and, if applicable, confounder-adjusted estimates and their precision (eg, 95% confidence interval). Make clear which confounders were adjusted for and why they were included | Results (‘Effects of age, coexisting conditions, sex, and vaccination on infection, ‘Effects of age, coexisting conditions, sex, and vaccination on severe, critical, or fatal COVID-19’), Figures 3-4, Table 2, & Figures S3-S4 and Table S3 in Supplementary Information |
|  |  | (b) Report category boundaries when continuous variables were categorized | Table 1 & Table S2 in Supplementary Information |
|  |  | (c) If relevant, consider translating estimates of relative risk into absolute risk for a meaningful time period | Not applicable |
| Other analyses | 17 | Report other analyses done—eg analyses of subgroups and interactions, and sensitivity analyses | Figures S3-S4 and Table S3 in Supplementary Information |
| Discussion | | | |
| Key results | 18 | Summarise key results with reference to study objectives | Discussion, paragraphs 1-6 |
| Limitations | 19 | Discuss limitations of the study, taking into account sources of potential bias or imprecision. Discuss both direction and magnitude of any potential bias | Discussion, paragraphs 7-10 |
| Interpretation | 20 | Give a cautious overall interpretation of results considering objectives, limitations, multiplicity of analyses, results from similar studies, and other relevant evidence | Discussion, paragraph 11 |
| Generalisability | 21 | Discuss the generalisability (external validity) of the study results | Discussion, paragraph 9 |
| Other information | | | |
| Funding | 22 | Give the source of funding and the role of the funders for the present study and, if applicable, for the original study on which the present article is based | Funding Information |

# **TABLE S2.** Baseline characteristics of Qatari participants.

| **Characteristics of study cohort** | **N (%)** |
| --- | --- |
|  | **N=42,455** |
| Median age at the start of follow-up (IQR)—years | 58.6 (53.6-65.3) |
| Age—years |  |
| 50-54 | 14,098 (33.2) |
| 55-59 | 9,991 (23.5) |
| 60-64 | 7,542 (17.8) |
| 65-69 | 3,481 (8.2) |
| 70-74 | 2,950 (6.9) |
| 75-79 | 2,052 (4.8) |
| 80+ | 2,341 (5.5) |
| Sex |  |
| Male | 19,387 (45.7) |
| Female | 23,068 (54.3) |
| Number of coexisting medical conditions |  |
| None | 9,035 (21.3) |
| 1 | 5,574 (13.1) |
| 2 | 5,875 (13.8) |
| 3 | 5,625 (13.2) |
| 4 | 5,643 (13.3) |
| 5 | 4,405 (10.4) |
| 6+ | 6,298 (14.8) |
| Vaccine dose^†^ |  |
| 0 | 9,548 (22.5) |
| 1 | 538 (1.3) |
| 2 | 20,894 (49.2) |
| 3 | 11,146 (26.3) |
| 4 | 323 (0.8) |
| 5 | 6 (0.01) |
| Testing frequency^‡^ |  |
| Low | 7,688 (18.1) |
| Intermediate | 23,898 (56.3) |
| High | 10,869 (25.6) |

IQR denotes interquartile range.

^†^Ascertained at time of censoring.

^‡^Low, intermediate, and high testers were defined as individuals with <1, 1-3, and ≥4 tests per person-year, respectively, during follow-up.

# **FIGURE S1.** Daily count of newly diagnosed SARS-CoV-2 infections between February 5, 2020 and June 15, 2023.


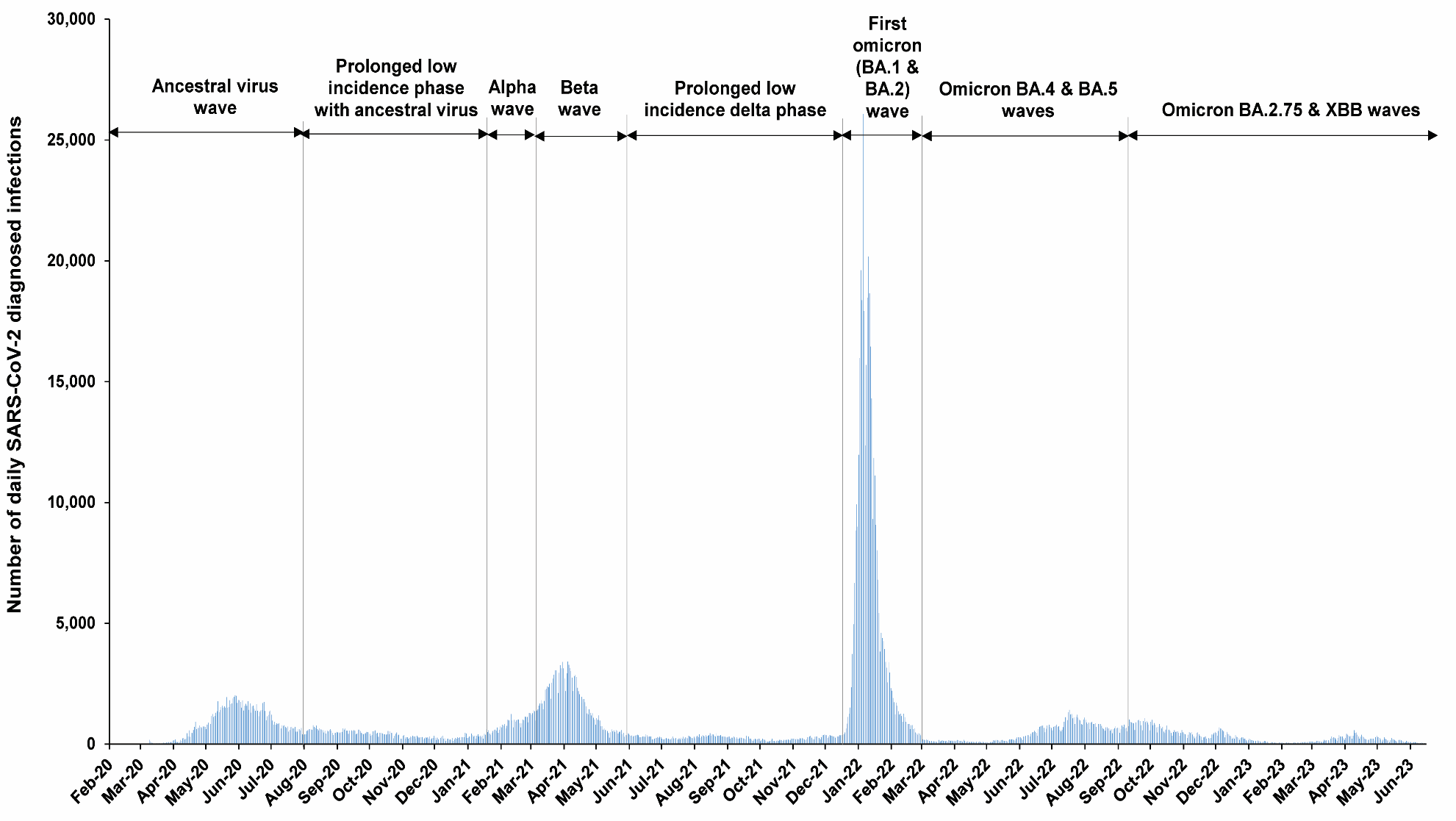


# **FIGURE S2.** Cumulative incidence of A) SARS-CoV-2 infection and B) severe, critical, or fatal COVID-19 disease among Qataris over the duration of the study.


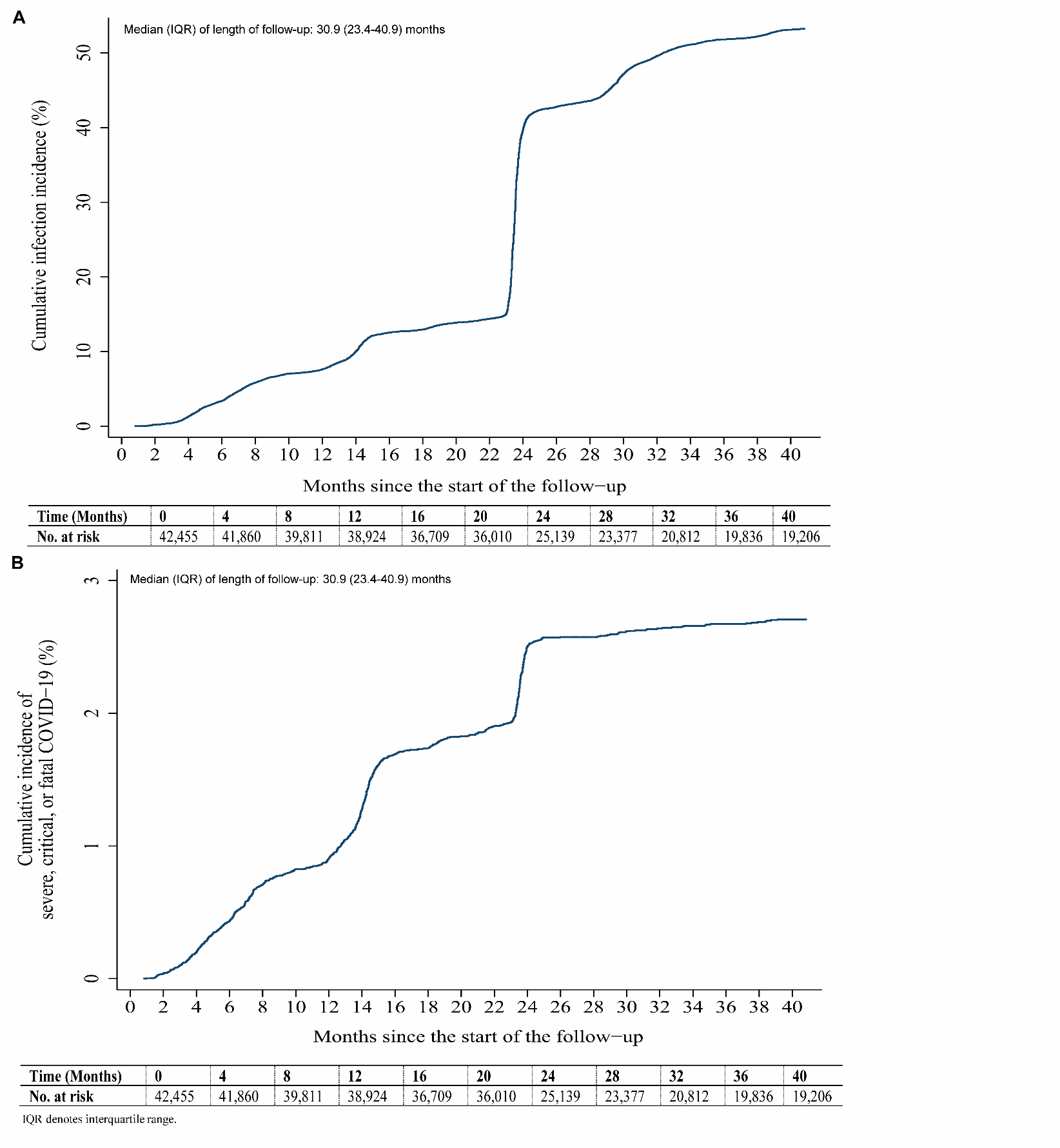


# **FIGURE S3.** Adjusted hazard ratios against SARS-CoV-2 infection among Qataris across A) age, B) number of coexisting conditions, C) sex, and D) vaccination dose status.

**
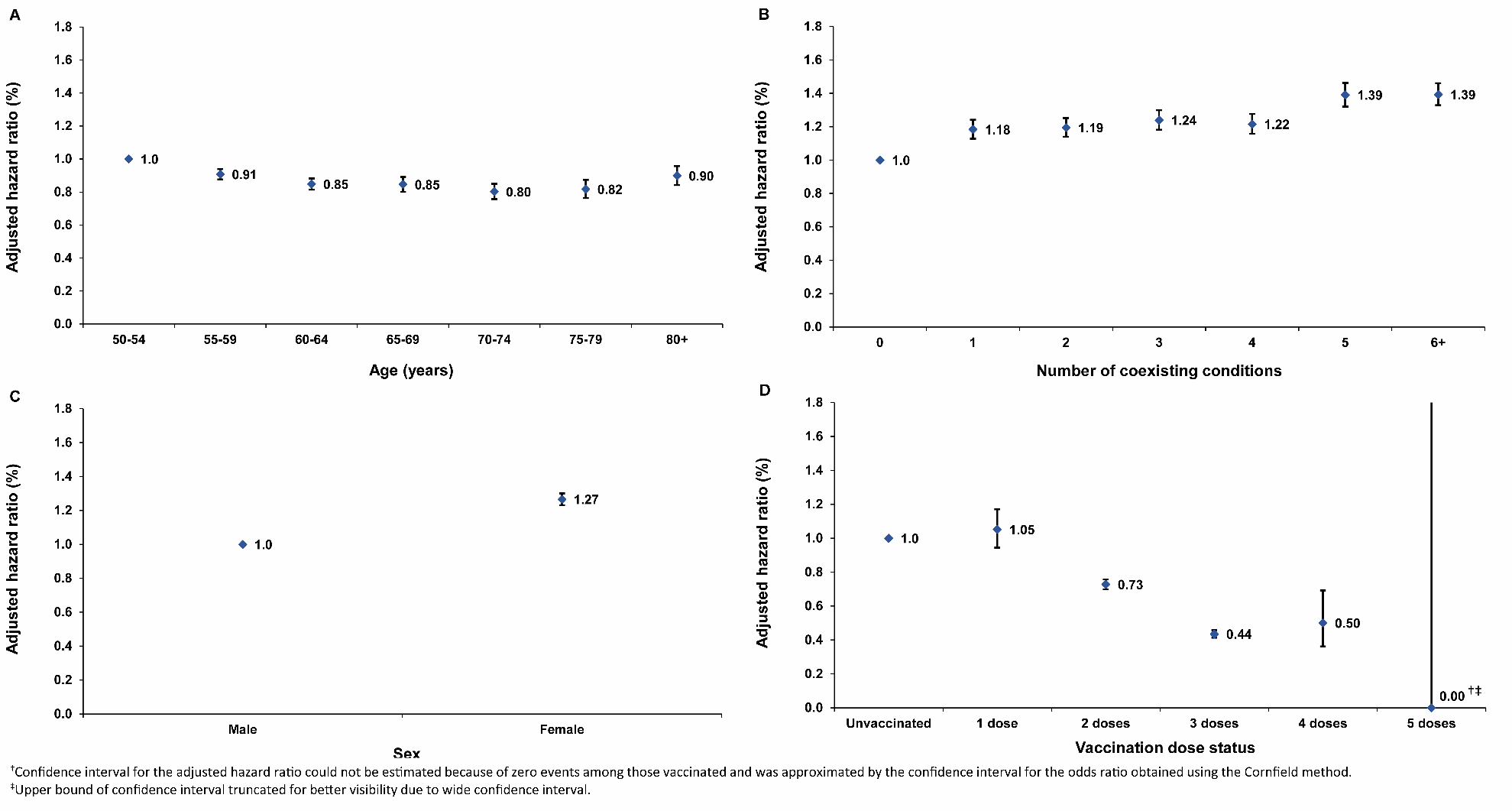
**

# **TABLE S3.** Adjusted hazard ratios against SARS-CoV-2 infection and against severe, critical, or fatal COVID-19 disease among Qataris across age, number of coexisting conditions, sex, and vaccination status.

| **Characteristics** | **Adjusted hazard ratio^†^ (95% CI)** | |
| --- | --- | --- |
|  | **Against SARS-CoV-2 infection** | **Against severe, critical, or fatal COVID-19^‡^** |
| **Age—years** |  |  |
| 50-54 | 1.00 | 1.00 |
| 55-59 | 0.91 (0.88-0.94) | 1.19 (0.95-1.47) |
| 60-64 | 0.85 (0.81-0.88) | 1.23 (0.98-1.54) |
| 65-69 | 0.85 (0.80-0.89) | 1.56 (1.20-2.01) |
| 70-74 | 0.80 (0.76-0.85) | 1.88 (1.47-2.41) |
| 75-79 | 0.82 (0.76-0.87) | 2.41 (1.87-3.11) |
| 80+ | 0.90 (0.84-0.96) | 3.46 (2.79-4.30) |
| **Number of coexisting medical conditions** |  |  |
| None | 1.00 | 1.00 |
| 1 | 1.18 (1.13-1.24) | 2.38 (1.67-3.39) |
| 2 | 1.19 (1.14-1.25) | 2.92 (2.09-4.08) |
| 3 | 1.24 (1.18-1.30) | 4.20 (3.07-5.76) |
| 4 | 1.22 (1.16-1.28) | 5.18 (3.82-7.03) |
| 5 | 1.39 (1.32-1.46) | 6.21 (4.56-8.45) |
| 6+ | 1.39 (1.33-1.46) | 7.14 (5.33-9.56) |
| **Sex** |  |  |
| Male | 1.00 | 1.00 |
| Female | 1.27 (1.23-1.30) | 0.70 (0.62-0.80) |
| **Vaccination status** |  |  |
| 0 | 1.00 | 1.00 |
| 1 | 1.05 (0.94-1.17) | 0.60 (0.43-0.82) |
| 2 | 0.73 (0.70-0.76) | 0.13 (0.11-0.16) |
| 3 | 0.44 (0.41-0.46) | 0.04 (0.03-0.07) |
| 4 | 0.50 (0.36-0.69) | 0.00 (0.00-0.64)^§^ |
| 5 | 0.00 (0.00-3.51) | 0.00 (0.00-35.21)^§^ |

CI denotes confidence interval COVID-19, coronavirus disease 2019, and SARS-CoV-2, severe acute respiratory syndrome coronavirus 2.

^†^Adjusted for 5-year age groups, number of coexisting medical conditions, sex, vaccination status, 10 nationality groups, and testing rate.

^‡^Severity,^24^ criticality,^24^ and fatality^24^ were defined according to the World Health Organization guidelines.

^§^CI for the adjusted hazard ratio could not be estimated because of zero events among those vaccinated and was approximated by the CI for the odds ratio obtained using the Cornfield method.

# **FIGURE S4.** Adjusted hazard ratios against severe, critical, or fatal COVID-19 disease among Qataris across A) age, B) number of coexisting conditions, C) sex, and D) vaccination dose status.

**
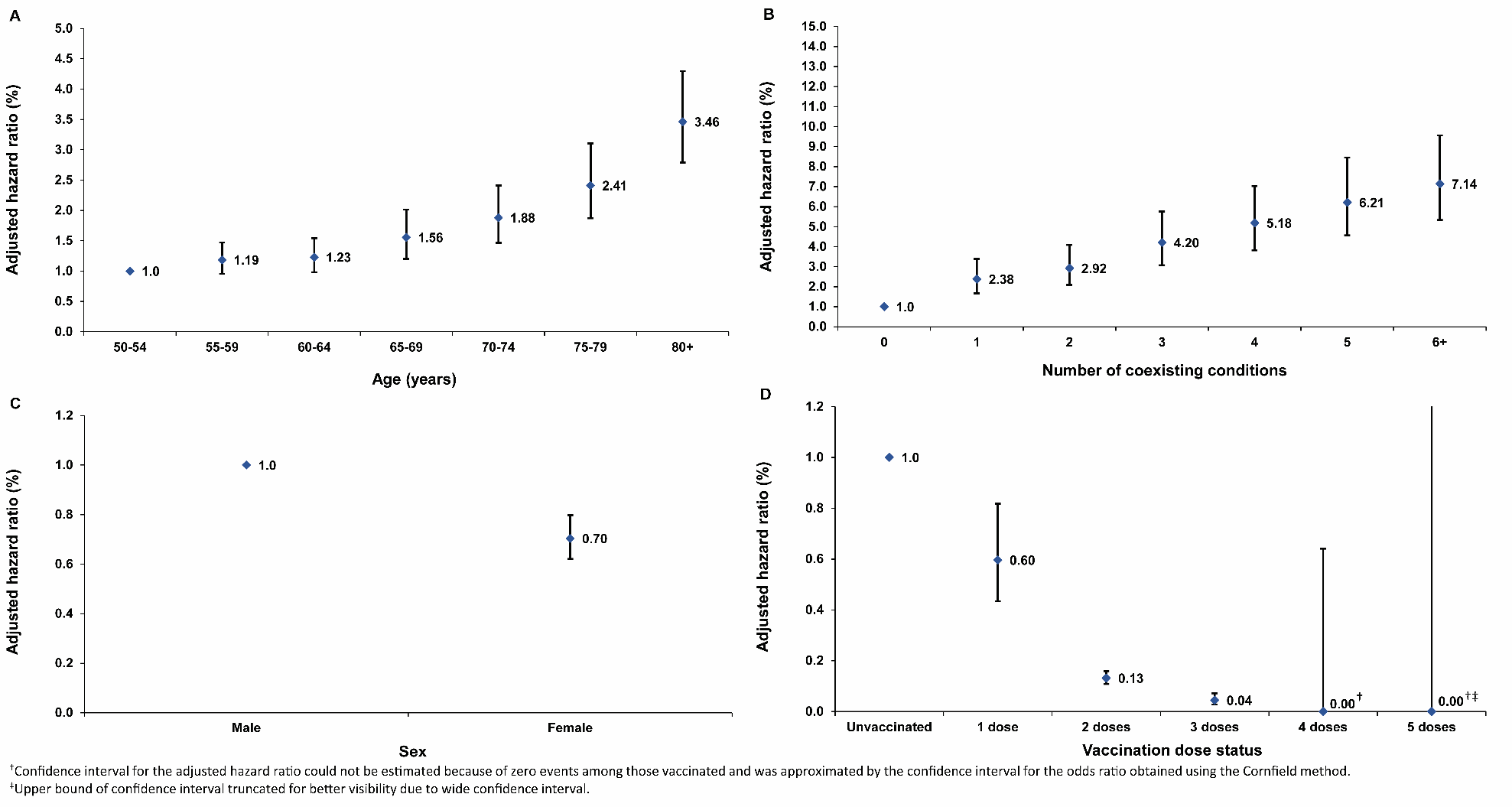
**

# **REFERENCES**

1. Altarawneh HN, Chemaitelly H, Ayoub HH, et al. Effects of Previous Infection and Vaccination on Symptomatic Omicron Infections. *N Engl J Med.* 2022;387(1):21-34.

2. Chemaitelly H, Tang P, Hasan MR, et al. Waning of BNT162b2 Vaccine Protection against SARS-CoV-2 Infection in Qatar. *N Engl J Med.* 2021;385(24):e83.

3. Chemaitelly H, Ayoub HH, AlMukdad S, et al. Bivalent mRNA-1273.214 vaccine effectiveness against SARS-CoV-2 omicron XBB* infections. *J Travel Med.* 2023.

4. Altarawneh HN, Chemaitelly H, Hasan MR, et al. Protection against the Omicron Variant from Previous SARS-CoV-2 Infection. *N Engl J Med.* 2022;386(13):1288-1290.

5. Chemaitelly H, Ayoub HH, Tang P, et al. Long-term COVID-19 booster effectiveness by infection history and clinical vulnerability and immune imprinting: a retrospective population-based cohort study. *Lancet Infect Dis.* 2023.

6. Chemaitelly H, Ayoub HH, Coyle P, et al. Protection of Omicron sub-lineage infection against reinfection with another Omicron sub-lineage. *Nat Commun.* 2022;13(1):4675.

7. Abu-Raddad LJ, Chemaitelly H, Ayoub HH, et al. Characterizing the Qatar advanced-phase SARS-CoV-2 epidemic. *Sci Rep.* 2021;11(1):6233.

8. Abu-Raddad LJ, Chemaitelly H, Ayoub HH, et al. Effect of mRNA Vaccine Boosters against SARS-CoV-2 Omicron Infection in Qatar. *N Engl J Med.* 2022;386(19):1804-1816.

9. Chemaitelly H, Faust JS, Krumholz H, et al. Short- and longer-term all-cause mortality among SARS-CoV-2- infected persons and the pull-forward phenomenon in Qatar. *medRxiv.* 2023:2023.2001.2029.23285152.

10. AlNuaimi AA, Chemaitelly H, Semaan S, et al. All-cause and COVID-19 mortality in Qatar during the COVID-19 pandemic. *BMJ Glob Health.* 2023;8(5).

11. Vogels C, Fauver J, Grubaugh N. Multiplexed RT-qPCR to screen for SARS-COV-2 B.1.1.7, B.1.351, and P.1 variants of concern V.3. dx.doi.org/10.17504/protocols.io.br9vm966. 2021(June 6, 2021).

12. Abu-Raddad LJ, Chemaitelly H, Butt AA, National Study Group for Covid Vaccination. Effectiveness of the BNT162b2 Covid-19 Vaccine against the B.1.1.7 and B.1.351 Variants. *N Engl J Med.* 2021;385(2):187-189.

13. Chemaitelly H, Yassine HM, Benslimane FM, et al. mRNA-1273 COVID-19 vaccine effectiveness against the B.1.1.7 and B.1.351 variants and severe COVID-19 disease in Qatar. *Nat Med.* 2021;27(9):1614-1621.

14. National Project of Surveillance for Variants of Concern and Viral Genome Sequencing. Qatar viral genome sequencing data. Data on randomly collected samples. <https://www.gisaid.org/phylodynamics/global/nextstrain/>. 2021; <https://www.gisaid.org/phylodynamics/global/nextstrain/>.

15. Benslimane FM, Al Khatib HA, Al-Jamal O, et al. One Year of SARS-CoV-2: Genomic Characterization of COVID-19 Outbreak in Qatar. *Front Cell Infect Microbiol.* 2021;11:768883.

16. Hasan MR, Kalikiri MKR, Mirza F, et al. Real-Time SARS-CoV-2 Genotyping by High-Throughput Multiplex PCR Reveals the Epidemiology of the Variants of Concern in Qatar. *Int J Infect Dis.* 2021;112:52-54.

17. Saththasivam J, El-Malah SS, Gomez TA, et al. COVID-19 (SARS-CoV-2) outbreak monitoring using wastewater-based epidemiology in Qatar. *Sci Total Environ.* 2021;774:145608.

18. El-Malah SS, Saththasivam J, Jabbar KA, et al. Application of human RNase P normalization for the realistic estimation of SARS-CoV-2 viral load in wastewater: A perspective from Qatar wastewater surveillance. *Environ Technol Innov.* 2022;27:102775.

19. Tang P, Hasan MR, Chemaitelly H, et al. BNT162b2 and mRNA-1273 COVID-19 vaccine effectiveness against the SARS-CoV-2 Delta variant in Qatar. *Nat Med.* 2021;27(12):2136-2143.

20. Chemaitelly H, Ayoub HH, AlMukdad S, et al. Duration of mRNA vaccine protection against SARS-CoV-2 Omicron BA.1 and BA.2 subvariants in Qatar. *Nat Commun.* 2022;13(1):3082.

21. Qassim SH, Chemaitelly H, Ayoub HH, et al. Effects of BA.1/BA.2 subvariant, vaccination and prior infection on infectiousness of SARS-CoV-2 omicron infections. *J Travel Med.* 2022;29(6).

22. Altarawneh HN, Chemaitelly H, Ayoub HH, et al. Protective Effect of Previous SARS-CoV-2 Infection against Omicron BA.4 and BA.5 Subvariants. *N Engl J Med.* 2022;387(17):1620-1622.

23. Chemaitelly H, Tang P, Coyle P, et al. Protection against Reinfection with the Omicron BA.2.75 Subvariant. *N Engl J Med.* 2023;388(7):665-667.

24. World Health Organization (WHO). Living guidance for clinical management of COVID-19. Aavailable from: <https://www.who.int/publications/i/item/WHO-2019-nCoV-clinical-2021-2>. Accessed on: February 27, 2023. 2023.

25. World Health Organization (WHO). International Guidelines for Certification and Classification (Coding) of COVID-19 as Cause of Death. Available from: <https://www.who.int/publications/m/item/international-guidelines-for-certification-and-classification-(coding)-of-covid-19-as-cause-of-death>. Accessed on: February 27, 2023. 2023.

26. Abu-Raddad LJ, Chemaitelly H, Coyle P, et al. SARS-CoV-2 antibody-positivity protects against reinfection for at least seven months with 95% efficacy. *EClinicalMedicine.* 2021;35:100861.

27. Abu-Raddad LJ, Chemaitelly H, Ayoub HH, et al. Introduction and expansion of the SARS-CoV-2 B.1.1.7 variant and reinfections in Qatar: A nationally representative cohort study. *PLoS Med.* 2021;18(12):e1003879.

28. Chemaitelly H, Bertollini R, Abu-Raddad LJ, National Study Group for Covid Epidemiology. Efficacy of Natural Immunity against SARS-CoV-2 Reinfection with the Beta Variant. *N Engl J Med.* 2021;385(27):2585-2586.

29. Altarawneh HN, Chemaitelly H, Ayoub HH, et al. Effects of previous infection, vaccination, and hybrid immunity against symptomatic Alpha, Beta, and Delta infections. *medRxiv.* 2023:2023.2004.2021.23288917.
